# Early fluid bolus in adults with sepsis in the emergency department: a systematic review, meta-analysis and narrative synthesis

**DOI:** 10.1186/s12873-021-00558-5

**Published:** 2022-01-11

**Authors:** Gladis Kabil, Steven A. Frost, Deborah Hatcher, Amith Shetty, Jann Foster, Stephen McNally

**Affiliations:** 1 Western Sydney University, School of Nursing and Midwifery, Locked bag 1797, Penrith, NSW 2751 Australia; 2 Department of Emergency, Westmead Hospital, Sydney, Australia; 3 South Western Sydney Nursing and Midwifery Research, Ingham Institute of Applied Medical Research, Sydney, Australia; 4 University of New South Wales, Sydney, Australia; 5 Westmead Institute for Medical Research, Westmead, Australia; 6 NSW Ministry of Health, New South Wales, Australia

**Keywords:** Sepsis, Fluid therapy, Barriers, Facilitators, Compliance

## Abstract

**Background:**

Early intravenous fluids for patients with sepsis presenting with hypoperfusion or shock in the emergency department remains one of the key recommendations of the Surviving Sepsis Campaign guidelines to reduce mortality. However, compliance with the recommendation remains poor. While several interventions have been implemented to improve early fluid administration as part of sepsis protocols, the extent to which they have improved compliance with fluid resuscitation is unknown. The factors associated with the lack of compliance are also poorly understood.

**Methods:**

We conducted a systematic review, meta-analysis and narrative review to investigate the effectiveness of interventions in emergency departments in improving compliance with early fluid administration and examine the non-interventional facilitators and barriers that may influence appropriate fluid administration in adults with sepsis. We searched MEDLINE Ovid/PubMed, Ovid EMBASE, CINAHL, and SCOPUS databases for studies of any design to April 2021. We synthesised results from the studies reporting effectiveness of interventions in a meta-analysis and conducted a narrative synthesis of studies reporting non-interventional factors.

**Results:**

We included 31 studies out of the 825 unique articles identified in the systematic review of which 21 were included in the meta-analysis and 11 in the narrative synthesis. In meta-analysis, interventions were associated with a 47% improvement in the rate of compliance [(Random Effects (RE) Relative Risk (RR) = 1.47, 95% Confidence Interval (CI), 1.25–1.74, *p*-value < 0.01)]; an average 24 min reduction in the time to fluids [RE mean difference = − 24.11(95% CI − 14.09 to − 34.14 min, *p* value < 0.01)], and patients receiving an additional 575 mL fluids [RE mean difference = 575.40 (95% CI 202.28–1353.08, *p* value < 0.01)]. The compliance rate of early fluid administration reported in the studies included in the narrative synthesis is 48% [RR = 0.48 (95% CI 0.24–0.72)].

**Conclusion:**

Performance improvement interventions improve compliance and time and volume of fluids administered to patients with sepsis in the emergency department. While patient-related factors such as advanced age, co-morbidities, cryptic shock were associated with poor compliance, important organisational factors such as inexperience of clinicians, overcrowding and inter-hospital transfers were also identified. A comprehensive understanding of the facilitators and barriers to early fluid administration is essential to design quality improvement projects.

**PROSPERO Registration ID:**

CRD42021225417.

**Supplementary Information:**

The online version contains supplementary material available at 10.1186/s12873-021-00558-5.

## Introduction

Sepsis is defined as a dysregulated immune response to infection, which if deteriorating to septic shock results in high mortality and morbidity [1]. It is a medical emergency now recognised as a global health priority [2, 3]. One of the key determinants of mortality is tissue hypoperfusion, which leads to multi-organ failure [4]. Therefore, restoration of cardiac output with early intravenous fluid bolus remains a mainstay of treatment in patients with septic shock [5]. In their landmark study, Rivers et.al [6] showed that early management of sepsis with appropriate fluids within the first 6 hours of presentation resulted in a 16% reduction in the risk of mortality.

The Surviving Sepsis Campaign has since provided several recommendations for sepsis management with goals to be achieved within the first 3 and 6 h [4]. The recommendations include administration of 30 mL/kg of intravenous fluid bolus for patients with hypotension; administration of antibiotics; obtaining blood culture and lactate [4]. Implementation of these guidelines over the last decades has resulted in an overall 16.7% decrease in mortality [7]. However, the Surviving Sepsis Guidelines have been revised periodically with ongoing changes to recommended time of initiation and completion of bundles varying from 6-h in 2005 to 1-h in 2018 [8] with the latest change to 3-h bundles in 2021 [5]. These changes have provoked debates among clinicians with some resistance to implementing these guidelines [8]. Concerns regarding fluid volume overload associated with injudicious use of intravenous fluids [9] have also resulted in some advocating for a conservative approach to fluid resuscitation [10]while others show benefit from early administration of fluid bolus [11]. However, it is important to note that these studies do not contradict the importance of early fluid administration but warn against unwarranted cumulative administration of intravenous fluids beyond the initial resuscitation phase. These clinical concerns and controversies are of particular relevance in settings like ED with time pressures and constant competing priorities.

Prompt recognition and management of sepsis in the emergency department remains an ongoing challenge. Compliance with critical interventions such as intravenous fluid bolus is reported to be poor [12]. Recently an increasing number of studies [13, 14] have examined the facilitators and barriers to the implementation of the Surviving Sepsis Campaign interventions with a subset of them analysing the time taken to initiate the first intravenous fluid bolus. However, the results of these studies have not been systematically identified and reported to summarise the overall benefit of the interventions to facilitate the timely administration of fluids in sepsis patients presenting to the emergency department. Therefore, we have undertaken a systematic review with meta-analysis and narrative synthesis [15, 16] to summarize the published literature investigating factors associated with early initiation of intravenous fluid bolus in patients presenting to the emergency department with sepsis.

This systematic review has two aims: (1) to describe the effectiveness of interventions implemented in the emergency department to improve compliance with early fluid bolus initiation, including time to, and the total volume of fluids administered by conducting a meta-analysis; and (2) to examine the non-interventional factors that may be barriers or facilitators to appropriate fluid administration using narrative synthesis.

## Methods

This systematic review has been conducted based on a priori protocol published in PROSPERO (ID: CRD42021225417), and planned, conducted and reported in accordance with the PRISMA statement [17]. We sought to conduct a mixed-method systematic review. However, the search results yielded only one qualitative study [18] that met the inclusion criteria. Therefore, a systematic review and meta-analysis of quantitative studies was conducted with a narrative synthesis of potential facilitators and barriers to appropriate fluid administration among adult septic patients presenting to the emergency department.

### Eligibility criteria

This review includes studies with experimental or quasi-experimental design that included adult patients (age > 17 years) presenting to the emergency department with sepsis, systemic inflammatory response syndrome (SIRS), severe sepsis, septic shock or a combination of any of these. Study designs included randomised controlled trials, before and after studies, prospective and retrospective cohort studies, case-control studies and analytical cross-sectional studies. Studies published before 2001 were excluded as the concept of early goal-directed therapy for sepsis was introduced in 2001. Studies conducted in settings other than the emergency department were excluded. The search was not restricted for language, however, only articles that were available in English were reviewed. We did not restrict studies if factors improving or increasing the time to first fluid bolus, were not reported as their main objectives, however all included studies provided data based upon either of these two factors.

For aim (1), any intervention or strategies implemented that influence early intravenous fluid bolus such as educational programs, sepsis alerts and sepsis protocols were included. Studies reporting the rate of compliance with intravenous fluid resuscitation, time to first fluid bolus, and volume of fluids administered were included. Studies with well-defined intervention and control groups were included in the meta-analysis. We did not impose restrictions based on the number of interventions implemented. For aim (2), studies exploring the influence of non-interventional factors such as over-crowding and inter-hospital transfers on compliance with early fluid bolus initiation were included in the narrative synthesis.

### Data sources and search strategy

The search strategy (Additional file 1) was developed in collaboration with two expert librarians and the search results were reviewed and verified. We systematically searched the electronic databases MEDLINE Ovid/PubMed, Ovid EMBASE, CINAHL, SCOPUS from inception through April 2021. The JBI and COCHRANE libraries were searched for related systematic reviews. Trove, ProQuest Dissertations, Google Scholar were used for grey-literature search. Reference lists from eligible studies were manually searched to identify additional studies.

### Study selection

After removing duplicates, two investigators (GK and SM) independently screened all identified titles and abstracts using COVIDENCE software for systematic reviews [19] and conflicts were resolved by a third investigator (SF). Articles not meeting the inclusion criteria were excluded, and the remaining were evaluated in full text. Disagreements were reconciled through discussion and consensus with all the investigators. Studies with interventions or strategies implemented that influence the compliance or time of administration and volume of intravenous fluid bolus were included in the meta-analysis. Studies reporting non-interventional factors were included in the narrative synthesis.

### Data extraction and quality assessment

Studies selected for retrieval were assessed by two independent reviewers (GK and SM) for methodological validity using standardised critical appraisal instruments from JBI SUMARI [20]. The results of the critical appraisal are presented as a table (Additional file 2). All studies regardless of their methodological quality were extracted and synthesised where possible.

The study characteristics data of all included studies was extracted using the standardised Joanna Briggs Institute data extraction tool in JBI SUMARI with details about the study population, study methods and outcomes of significance to the review objective (Additional file 3). The information included in data extraction for studies included in the meta-analysis are presented in Table 1. The primary outcome of interest was the time to and volume of initial fluid bolus administration or the rates of compliance with the internationally accepted Surviving Sepsis Campaign guidelines.

**Table 1:** Data Extraction Information for Studies included in the Meta-analysis

S. No	Information
1	Author
2	Year of publication
3	Study design
4	Emergency department type
5	Number of patients enrolled in the control group
6	Number of patients in the intervention group
7	Number compliant with early intravenous fluid bolus in control group
8	Number complaint with early intravenous fluid bolus in intervention group
9	Time to administration of first fluid bolus in control group
10	Time to administration of first fluid bolus in intervention group
11	Volume of fluids received by patients in control group
12	Volume of fluids received by patients in intervention group
13	Whether or not the studied intervention had any improvement in compliance with time and volume of initial fluid bolus administered
14	Number of interventions implemented

### Data analysis

The meta package V4.17–0 [21] in R statistical language [22]was used to conduct the meta-analysis. We summarised the effectiveness of the interventions on compliance, time to administration and volume administered in a meta-analysis. We grouped the studies based on outcomes reported as compliance rate. We have reported both the random-effects model (DerSimonian-Laird estimator) and fixed-effects model (Mantel-Haenszel estimator). For continuous variables (time to fluids and volume), the mean and standard deviations (SD) or the median and interquartile range (IQR) were presented. For meta-analysis of these continuous outcomes, if only median and IQR were reported, mean and SD were derived using the method suggested by Luo and Shi [23–26]. Heterogeneity between studies was assessed using an *I*^*2*^ statistic and a *p*-value < 0.1 was chosen to represent evidence of statistical heterogeneity. Publication bias was assessed by inspection of funnel plots and asymmetry was assessed using the regression test suggested by Egger (a *p*-value < 0.1 was considered as evidence of funnel plot asymmetry) [27].

The narrative synthesis was undertaken using the Guidance for Systematic Reviews [28, 29]. We identified factors such as overcrowding, inter-hospital transfer and failure to recognise sepsis reported in the studies that were not suitable for a meta-analytical approach. To synthesise all these factors, we tabulated the data from these studies and used textual description of the identified factors. We then regrouped the factors based on whether they were found to be barriers, facilitators or factors that had no influence on the early initiation of intravenous fluid bolus in sepsis along with any additional recommendations reported in the studies (Table 2).

**Table 2:** Narrative Synthesis with description of factors influencing early initiation of fluid bolus in sepsis

Study Name	Sample size (n)	Facilitators identified	Barriers identified	Compliance Rate	Factors that had no influence	Recommendations
Baldwin (2008) [30]	32	Near patient lactate testing	Underestimating the severity of sepsis; incomplete triage data hindering prompt diagnosis; first assessment done by very junior doctors.	53%		100% completion of triage vital signs; review by middle grade doctors within first 30 min; training nurses and doctors.
Kang (2012) [31]	317	Care by board-certified emergency physicians; nurses with > 3 yrs. experience	Patients with cryptic shock, higher serum lactate levels or without hyperthermia; care by junior resident doctors	256 (80.8%)	Overcrowding; sex- based differences of the treating physician	Interventions focussing on the identified barriers
Shin (2012)	770		ED overcrowding	81.9%		Multidisciplinary response team; effective bed management
Gray (2013) [32]	626		Difficulty recognising sepsis; clinical reliance on development of hypotension	48%		Pre-hospital sepsis screening criteria
Wang (2013) [33]	195		Survey response to why IV fluid challenge was not achieved: 41% unsure; 59% didn’t think it was needed. Knowledge, attitude and behavioural barriers.	27% (Control group)		
Faine (2015) [34]	193		Interhospital transfers from regional hospitals; inadequacy of emergency trained physicians in rural hospitals; clinical deterioration of patient during transfer.	54% (Patients transferred from regional hospitals)		Use of telemedicine
De Groot (2017) [35]	1732	Treatment commenced in ED patients in earlier stages of sepsis				Emphasis on treatment in patients with and without organ failure in sepsis
Gaieski (2017) [36]	2913		Time of presentation of patients to ED (between 07:00–19:00 less likely to receive fluids within 1 h compared to presenting after-hours); overcrowding, increased occupancy rate and patient hours in ED			Appropriate staffing and patient flow in ED
Morr (2017) [37]	487				Correctness of exact classification of sepsis- SIRS, severe sepsis, recognised or unrecognised sepsis	
Le Conte (2017) [38]	130		Advanced age; cardiac co-morbidities; delay in sepsis recognition; ED overcrowding;	25 (19%) received fluid challenge, Mean time to administration: 10 ± 27 min		Multidisciplinary quality improvement programme with simple guidelines, electronic alerts; qSOFA score measurement
Deis (2018) [39]	5631		Patients without an ICD sepsis diagnosis code despite similar baseline organ dysfunction	10.6% for patients without a sepsis diagnosis code; 19.6% for patients with a diagnosis code		Education and quality improvement outcomes

### Assessing temporal trend

To examine any potential impact of the changes to the Surviving Sepsis Guidelines over the years, cumulative meta-analysis [40] was performed using the year the studies were commenced reflecting the guideline changes during the study period. In addition, meta regression was done using the year studies commenced as a covariate.

## Results

**Fig. 1:**
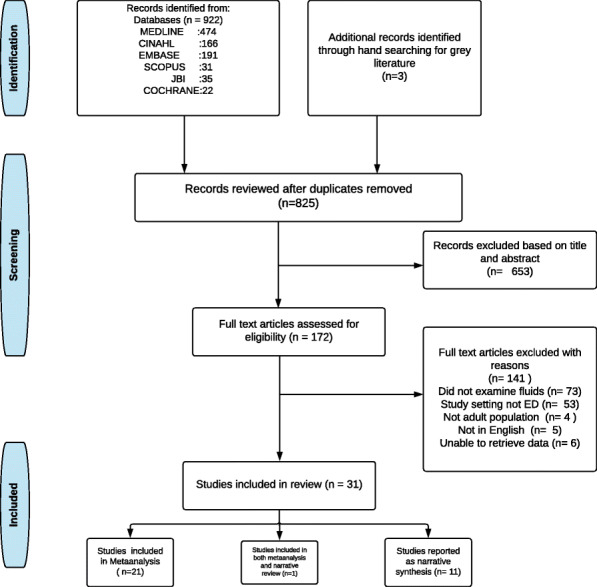
PRISMA flowchart of study inclusion

The process of identifying studies to be included in the review at various stages are presented as a PRISMA flow-diagram in Fig. 1. The initial electronic search from databases identified 925 potential articles, of which 100 articles were identified as duplicates. Titles and abstracts of 825 articles were screened for eligibility, of which 172 articles were retained for full-text review. After applying the inclusion and exclusion criteria, 31 articles were retained. Twenty-one of these studies [33, 41–60] included interventions and reported the effectiveness of these interventions and were subsequently included in a meta-analysis. Eleven studies that reported non-interventional factors are presented as a narrative synthesis. One [33] of the eleven studies reported both intervention and explored the non-interventional factors impacting the compliance using a survey and has therefore been included in both the meta-analysis and the narrative synthesis. Among the studies included in the meta-analysis, we observed variations in the definitions used to define sepsis and septic shock including Sepsis 2 definition, Sepsis 3 definition, International Classification of Diseases (ICD) Codes 9 and 10 (see Additional file 3). Of the studies included in the analysis of time to first fluid bolus, most studies used time of arrival at triage as the time zero while two studies [54, 59], have used time of diagnosis of sepsis as part of the intervention as the time zero (see Additional file 3).

### Meta-analysis

The studies included in the meta-analysis were analysed based on data reported for three specific outcome measures: (1) compliance with early fluid bolus administration within the protocol recommended time; (2) time to administration of initial fluid bolus; and (3) volume of fluids administered within the protocol recommended time.

#### Compliance with early fluid bolus administration

**Fig. 2:**
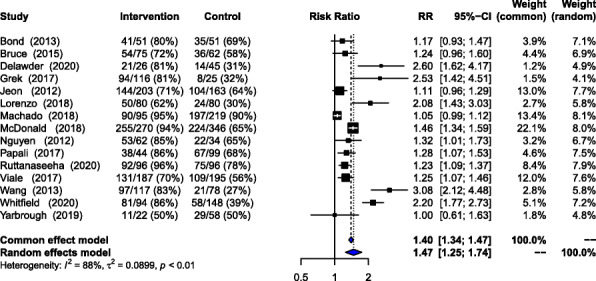
Association between intervention and compliance with early initiation of intravenous fluid bolus

Fifteen studies enrolling 1538 patients in the intervention group and 1969 patients in the control group investigated the effectiveness of the interventions on the rate of compliance with the initiation of 20-30 mL/Kg of intravenous fluid bolus within 3–6 h of presenting at triage in the emergency department. Individual study and summary estimates of the comparison of rates of compliance during the intervention and control period are presented in Fig. 2. A random effects summary meta-analysis estimated a 47% improvement in the rate of compliance with early fluid bolus initiation during the intervention period compared to the control period (Random Effects (RE) Relative Risk (RR) = 1.47, 95% Confidence Interval (CI), 1.25–1.74, *p*-value < 0.01).

#### Time to initiation of first fluid bolus

**Fig. 3:**
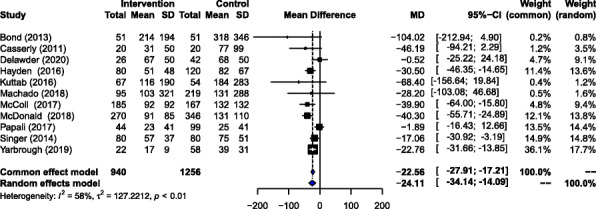
Association between intervention and time of initiation of first intravenous fluid bolus administration

Eleven studies evaluated the impact of the interventions on the time of initiation of the first intravenous fluid bolus (Fig. 3). A total of 940 patients were enrolled in the intervention group and 1256 patients in the control group. The pooled (RE) estimate of the mean difference in time was − 24.11 min (95% CI − 14.09 to − 34.14 min, *p*-value < 0.01), indicating an average 24 min reduction in the time to fluid resuscitation between the intervention and control groups.

#### Volume of fluids administered

**Fig. 4:**
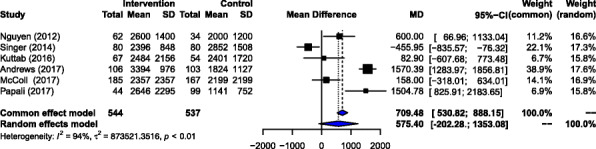
Association between intervention and volume of fluids administered within the protocol recommended time

Six studies with 537 patients in the control group and 544 patients in the intervention group were evaluated for the difference in the volume of fluids administered to patients who received the intervention (Fig. 4). The pooled (RE) effect size was a mean of 575.40 mL (95% CI 202.28–1353.08 mL, *p* value < 0.01), indicating that patients received an average additional 575 mL within the protocol recommended time as a result of the interventions.

#### Publication bias

All studies included in the three outcome groups of meta-analysis were assessed for publication bias using visual inspection of the funnel plots (Additional file 4). Among the included studies, except for the volume of fluids studies, there was no evidence of significant publication bias on visualisation of the funnel plots.

#### Meta-regression

Considerable statistical heterogeneity was found in studies reporting compliance and volume of fluids administered in the meta-analysis with *I*^*2*^ values of 90 and 94% respectively with *p* values < 0.01 and substantial heterogeneity found in the studies analysing time to fluids (*I*^2^58%, p value < 0.01). No statistically significant regression co-efficients were found using the mortality as a covariate in studies reporting compliance and volume of fluids, however in studies reporting time to fluids, mortality explained 45.4% of the heterogeneity (R^2^ = 45.4%, QE = 7.25, p value < 0.404). Using the number of interventions used as a covariate, with more than one intervention treated as a bundle of interventions, in the studies reporting the volumes of fluids administered, 75.4% of the heterogeneity was accounted for with the number of interventions (R^2^ 75.4%, QE = 11.8, p value 0.008). However, the heterogeneity in the studies analysing time to fluids and compliance could not be explained using the number of interventions. Key results of the meta regression are presented in Additional file 5.

#### Temporal trend

The results of the cumulative meta-analysis are presented in Additional file 6. Visual exploration of temporal trends based on the year the studies commenced reflecting the changes to the guidelines used in the studies did not show a significant relationship with the compliance with early fluid bolus administration, time to administration of fluids and volume of fluids administered. Meta-regression performed using year of study as a covariate did not show significant regression co-efficients (see Additional file 5).

### Narrative synthesis

Of the eleven studies included in the narrative synthesis, eight studies reported the proportion of patients with sepsis who received early intravenous fluid bolus in the emergency department with a total of 13,026 patients included in the studies. The pooled estimate is 0.48 (95% CI 0.24–0.72), indicating that the average compliance of patients who received early fluid bolus is 48%. Most of these studies were conducted as retrospective chart analysis or audit. The factors identified as barriers and facilitators are summarised in Table 2.

#### Identifiable barriers to early fluid bolus initiation in sepsis

Barriers to the early initiation of intravenous fluids was reported in eight out of the eleven studies. The patient-related factors reported were presentation with cryptic shock, those with higher serum lactate level, patients without hyperthermia on presentation, and those who have an advanced age, and cardiac co-morbidities [30, 31]. Most studies have reported non-patient related factors as barriers. Improper diagnosis of sepsis such as underestimating the severity of sepsis, difficulty and delay in recognising sepsis, and clinical reliance on development of hypotension to begin treatment have been repeatedly reported [32, 34, 38]. Experience of the treating healthcare professional has been identified as an inhibiting factor when inexperienced junior physicians and those without emergency training care for patients during the initial hours [30, 31, 34]. Assessment errors such as incomplete triage vital signs data and incorrect allocation of sepsis specific International Classification of Diseases (ICD) codes are reported to cause delays in commencing fluids [30, 39]. In contrast, Morr et al., report that correctness of the classification of sepsis and its severity had no impact on early initiation of fluid bolus [37]. Three studies have reported that overcrowding in the emergency department and its associated factors such as increased occupancy rate, increased patient hours and patients presenting between 0700 and 1900 h was strongly associated with delays in initiating fluid bolus [36, 38, 61]. However, the retrospective study by Kang et al., shows that over-crowding had no impact on the compliance rate [31]. Other reported factors include delays caused due to inter-hospital transfers from regional to referral centres associated with natural disease progression enroute [34] and knowledge, attitude and behavioural barriers of the healthcare professionals [33].

#### Factors improving early fluid bolus administration in sepsis

Three studies reported factors identified to improve early fluid bolus administration. Access to near patient lactate testing [31], treatment commenced in the earlier stages of sepsis without organ failure [35] and care provided by emergency trained physicians and nurses with more than 3 years of clinical experience [30] have been found to be associated with improved compliance with early fluid bolus administration.

#### Recommendations for future practice

Nine studies have suggested recommendations to improve compliance with early fluid administration. All nine studies recommend interventions specifically targeting the identified barriers which include 100% completion of triage vital signs data [30], pre-hospital sepsis screening [32], use of telemedicine [34], and use of assessment tools like qSOFA [38].Two studies have recommended appropriate staffing and bed flow to manage overcrowding [36, 61]. Quality improvement projects involving multidisciplinary teams and electronic alerts along with professional development on sepsis management for nurses and physicians have also been suggested by five studies [30, 31, 38, 39, 61].

## Discussion

Our systematic review, meta-analysis and narrative synthesis have identified a number of factors associated with the early initiation of intravenous fluid bolus in patients presenting with sepsis to the emergency department. Overall, interventions aimed at improving the management of sepsis in the emergency department increased compliance with early fluid bolus administration by 47%, reduced the time to fluid administration by an average 24 min, and increased the volume of fluids given by 575 mL. Importantly, this improvement was seen across a variety of emergency departments, worldwide. However, we found that it was uncommon for studies to specifically explore barriers to the implementation of interventions that improve the management of sepsis in the emergency department.

Our findings are consistent with a previous meta-analysis reporting improved compliance with the entire surviving sepsis bundle across various settings [62]. Most of the studies included in this meta-analysis analysed fluid administration as part of assessing the outcomes of the entire surviving sepsis bundle. Several of these studies showed that the proportion of improvement with fluid bolus administration was still lower compared with the other components of the surviving sepsis bundle such as antibiotics administration and lactate measurement [41–49]. Quality improvement programs specifically tailored for each element of the surviving sepsis bundle and targeting their approach towards fluid administration would be necessary to improve compliance with individual elements of the bundle. An understanding of factors that specifically influence compliance with early fluid administration is necessary to design suitable performance improvement measures.

The interventions implemented varied from educational, to process change measures such as a multidisciplinary sepsis program. We did not investigate the association between the interventions as single/bundled. However, meta regression showed interventions implemented as a bundle had a significant impact on the volume of fluids received by patients. However, this effect was not observed in the studies reporting the rate of compliance and time to initial fluids. Regardless of the type and number of interventions, the overall improvement in early fluid bolus indicates that a general increase in awareness and focus on fluid management in sepsis management improves performance. Although the guideline changes over the years have not shown significant impact on the compliance with early fluid bolus, time to fluid bolus administration, and the volume of fluids administered in this study, the impact the frequently changing guidelines could have on clinical practice variations and clinician decision-making cannot be disregarded warranting further qualitative research. Though all the studies that were meta-analysed have analysed the effectiveness of the interventions, only one study [33] reported the factors associated with initiation of an early fluid bolus. Consideration of the factors influencing the interventions is a key strategy to developing sustainable interventions in the clinical settings [63].

According to the results from the narrative synthesis, the baseline compliance with early fluid bolus administration is considerably low at 48%. This is congruent with the compliance rates for fluid resuscitation reported in studies conducted in other settings such as the wards and intensive care units with less than half the patients receiving fluids within the protocol recommended time [64, 65].Only a few studies have reported the barriers and facilitators specific to initial fluid bolus administration and we have included those in the narrative synthesis. While some of the barriers reported are similar to those reported regarding barriers to the implementation of the entire surviving sepsis bundle [66] such as insufficient sepsis training and knowledge [33], our analysis has found factors specifically impacting initial fluid bolus administration. These include patient-related factors such as advanced age and cardiac co-morbidities supporting recommendations for cautious fluid administration in patients within such subgroups [65, 67, 68]. Clinician associated issues such as inexperience, relying on hypotension as a clinical sign to commence fluid bolus and inaccurate diagnosis including incomplete triage vital signs, warrants educational interventions specifically tailored to knowledge deficits regarding fluid resuscitation in sepsis. On the other hand, organisational factors such as over-crowding and inter-hospital transfers require a more systemic approach. Although non-interventional factors such as treatment by experienced clinicians and treating patients in earlier stages of sepsis have been reported to facilitate early fluid bolus, the overall low rate of compliance from the pooled estimate in the narrative synthesis suggests that further studies are required to explore the facilitators and barriers to early fluid bolus.

Our study has several limitations. All except one [42] included study were observational in nature and therefore cannot account for casual relationships. In addition, the effect of other confounding variables such as severity of illness between the intervention and control group patients could not be identified from the available data. The studies have been conducted across different countries and differing emergency settings including resource limited settings [36, 37]which makes generalisability of these findings difficult. Substantial heterogeneity among studies means the pooled results need to be interpreted with caution. We conducted meta regression to explore the possible sources for heterogeneity, however, we cannot account for the influence of unmeasured sources. Despite our intention to include qualitative research using a mixed-method approach, lack of qualitative studies meeting the inclusion criteria prevented us from conducting a mixed-method review. Future qualitative studies exploring experiences of healthcare workers regarding fluid resuscitation in sepsis could provide wider range of sources. A number of studies exploring surviving sepsis bundles did not have data relating specifically to early fluid bolus initiation limiting the number of studies that could be included in the analysis. Finally, although we conducted an extensive electronic database search and review using a systematic approach, we cannot exclude the possibility of missing studies.

## Conclusion

Despite the limitations, our study offers a comprehensive understanding of the factors influencing early fluid bolus in sepsis. Our findings show that the overall compliance rate with early fluid bolus administration in adult patients with sepsis presenting to the emergency department is less than optimal. However, performance improvement initiatives significantly improve compliance with early fluid bolus and improves time to and volume of fluids administered. In this study, we have not only focussed on the effectiveness of interventions, we have also explored the facilitators and barriers specifically impacting early fluid resuscitation. Recognition of specific factors will assist in designing suitable performance improvement initiatives incorporating tailored measures targeting fluid administration rather than a “One size fits all” approach. Future studies using qualitative approach are required to further understand subjective factors influencing early fluid bolus.

## Supplementary Information

**Additional file 1.**

**Additional file 2.**

**Additional file 3.**

**Additional file 4.**

**Additional file 5.**

**Additional file 6.**

## Data Availability

Not Applicable.

## Abbreviations

ICD: International Classification of Diseases
PRISMA: Preferred Reporting Items for Systematic Reviews and Meta-Analyses
PROSPERO: The International Prospective Register of Systematic Reviews
SIRS: Systemic Inflammatory Response Syndrome
qSOFA: quick Sequential Organ Failure Assessment
