# Supplementary material for: Early fluid bolus in adults with sepsis in the emergency department: a systematic review, meta-analysis and narrative synthesis
Source: BMC Emerg Med. 2022 Jan 11;22:3. doi: 10.1186/s12873-021-00558-5 (PMC8753824; doi:10.1186/s12873-021-00558-5)
Supplement: Supplementary file 1 — Additional file 1. [file 12873_2021_558_MOESM1_ESM.docx]

**Additional File 1**

| **MEDLINE Complete Search Strategy** | | |
| --- | --- | --- |
| **Platform: OVID** | | |
| **Search Date: 10/12/2020 to 23/04/2021** | | |
| **ID** | **Search expression** | **Number of results** |
| 1 | exp Sepsis/ | 126995 |
| 2 | Severe Sepsis.mp. | 8734 |
| 3 | exp Shock, Septic/ | 23087 |
| 4 | Septicemia.mp. | 15062 |
| 5 | Septicaemia.mp. | 6463 |
| 6 | Pyohemia.mp. | 10 |
| 7 | Pyaemia.mp. | 133 |
| 8 | Pyemia.mp. | 71 |
| 9 | exp Systemic Inflammatory Response Syndrome/ | 132145 |
| 10 | SIRS.mp. | 5793 |
| 11 | septic*.mp. | 90708 |
| 12 | 1 or 2 or 3 or 4 or 5 or 6 or 7 or 8 or 9 or 10 or 11 | 182103 |
| 13 | exp Emergency Service, Hospital/ | 83407 |
| 14 | emergency department*.mp. | 101055 |
| 15 | ED.mp. | 83942 |
| 16 | exp Emergencies/ | 41284 |
| 17 | emergency.mp. | 333397 |
| 18 | emergency ward*.mp. | 1183 |
| 19 | Emergency Room*.mp. | 20502 |
| 20 | ER.mp. | 97346 |
| 21 | Emergency Unit*.mp. | 2296 |
| 22 | (accident and emergency).mp. [mp=title, abstract, original title, name of substance word, subject heading word, floating sub-heading word, keyword heading word, organism supplementary concept word, protocol supplementary concept word, rare disease supplementary concept word, unique identifier, synonyms] | 10540 |
| 23 | A&E.mp. | 25217 |
| 24 | 13 or 14 or 15 or 16 or 17 or 18 or 19 or 20 or 21 or 22 or 23 | 522212 |
| 25 | EGDT.mp. | 201 |
| 26 | Goal Directed Therapy.mp. | 1117 |
| 27 | Early Resuscitation.mp. | 431 |
| 28 | Goal Oriented Therapy.mp. | 46 |
| 29 | EGOT.mp. | 65 |
| 30 | exp Evidence-Based Practice/ | 90233 |
| 31 | EBP.mp. | 8590 |
| 32 | Sepsis clinical Pathway*.mp. | 2 |
| 33 | Sepsis Pathway *.mp. | 0 |
| 34 | Protocol*.mp. | 626280 |
| 35 | pathway*.mp. | 1288205 |
| 36 | Early Goal Directed Strategy.mp. | 0 |
| 37 | exp Resuscitation/ | 98532 |
| 38 | Surviving sepsis.mp. | 796 |
| 39 | Bundle*.mp. | 75424 |
| 40 | Bundled Care.mp. | 97 |
| 41 | Bundled treatment.mp. | 6 |
| 42 | exp Guideline/ | 35586 |
| 43 | exp Guideline Adherence/ | 33293 |
| 44 | exp Fluid Therapy/ | 20734 |
| 45 | exp Fluid Therapy/nu [Nursing] | 465 |
| 46 | Fluid*.mp. | 637465 |
| 47 | Intravenous fluid*.mp. | 5857 |
| 48 | exp Infusions, Intravenous/ | 55589 |
| 49 | exp Infusions, Intravenous/nu [Nursing] | 844 |
| 50 | IV fluid*.mp. | 1525 |
| 51 | Early fluid*.mp. | 265 |
| 52 | fluid resuscitation.mp. | 5401 |
| 53 | Goal Directed Fluid Therapy.mp. | 295 |
| 54 | fluid bolus.mp. | 548 |
| 55 | fluid administration.mp. | 2409 |
| 56 | appl*.mp. | 2435716 |
| 57 | implement*.mp. | 542933 |
| 58 | framework.mp. | 280047 |
| 59 | 25 or 26 or 27 or 28 or 29 or 30 or 31 or 32 or 33 or 34 or 35 or 36 or 37 or 38 or 39 or 40 or 41 or 42 or 43 or 44 or 45 or 46 or 47 or 48 or 49 or 50 or 51 or 52 or 53 or 54 or 55 or 56 or 57 or 58 | 5518849 |
| 60 | Barrier*.mp. | 334200 |
| 61 | exp Compliance/ | 4011 |
| 62 | Management.mp. | 1361106 |
| 63 | Delay*.mp. | 577072 |
| 64 | Early Management.mp. | 2930 |
| 65 | nurs* initiated.mp. | 429 |
| 66 | nur*driven.mp. [mp=title, abstract, original title, name of substance word, subject heading word, floating sub-heading word, keyword heading word, organism supplementary concept word, protocol supplementary concept word, rare disease supplementary concept word, unique identifier, synonyms] | 0 |
| 67 | emergency nur*.mp. [mp=title, abstract, original title, name of substance word, subject heading word, floating sub-heading word, keyword heading word, organism supplementary concept word, protocol supplementary concept word, rare disease supplementary concept word, unique identifier, synonyms] | 8604 |
| 68 | exp "Attitude of Health Personnel"/ | 161881 |
| 69 | exp Patient Compliance/ | 80186 |
| 70 | facilitat*.mp. | 571112 |
| 71 | exp Time Factors/ | 1205357 |
| 72 | time*.mp. | 4848896 |
| 73 | Barriers terms combined | 7094009 |
| 74 | exp Time Factors/ | 1205357 |
| 75 | time*.mp. | 4848896 |
| 76 | 60 or 61 or 62 or 63 or 64 or 65 or 66 or 67 or 68 or 69 or 70 or 71 or 72 or 73 or 74 or 75 | 7094009 |
| 77 | 12 and 24 and 59 and 76 | 1172 |
| 78 | limit 74 to (yr="2001 -Current" and "all adult (19 plus years)") | 474 |
