# Supplementary material for: Early fluid bolus in adults with sepsis in the emergency department: a systematic review, meta-analysis and narrative synthesis
Source: BMC Emerg Med. 2022 Jan 11;22:3. doi: 10.1186/s12873-021-00558-5 (PMC8753824; doi:10.1186/s12873-021-00558-5)
Supplement: Supplementary file 2 — Additional file 2. [file 12873_2021_558_MOESM2_ESM.docx]

### **Additional File 2- Critical Appraisal**

### Critical Appraisal Results

#### Table: Analytical Cross-Sectional Study

| **Citation** | **Q1** | **Q2** | **Q3** | **Q4** | **Q5** | **Q6** | **Q7** | **Q8** |
| --- | --- | --- | --- | --- | --- | --- | --- | --- |
| Baldwin LN, Smith SA, Fender V, Gisby S, Fraser J. 2008. | Y | Y | Y | Y | U | U | Y | U |
| % | 100.0 | 100.0 | 100.0 | 100.0 | 0.0 | 0.0 | 100.0 | 0.0 |

#### Table: Case Control Study

| **Citation** | **Q1** | **Q2** | **Q3** | **Q4** | **Q5** | **Q6** | **Q7** | **Q8** | **Q9** | **Q10** |
| --- | --- | --- | --- | --- | --- | --- | --- | --- | --- | --- |
| Bond CM, Djogovic D, Villa-Roel C, Bullard MJ, Meurer DP, Rowe BH. 2013. | Y | Y | Y | Y | Y | Y | N/A | Y | N/A | Y |
| % | 100.0 | 100.0 | 100.0 | 100.0 | 100.0 | 100.0 | 0.0 | 100.0 | 0.0 | 100.0 |

#### Table: Cohort Study

| **Citation** | **Q1** | **Q2** | **Q3** | **Q4** | **Q5** | **Q6** | **Q7** | **Q8** | **Q9** | **Q10** | **Q11** |
| --- | --- | --- | --- | --- | --- | --- | --- | --- | --- | --- | --- |
| De Groot B, Struyk B, Najafi R, Halma N, Pelser L, Vorst D, et al. 2017. | Y | Y | Y | Y | Y | Y | Y | N/A | N/A | N/A | Y |
| Deis AS, Whiles BB, Brown AR, Satterwhite CL, Simpson SQ. 2018. | N | Y | Y | N | N/A | Y | Y | N/A | N/A | N/A | Y |
| Faine BA, Noack JM, Wong T, Messerly JT, Ahmed A, Fuller BM, et al. 2015. | Y | Y | Y | Y | N/A | Y | Y | N/A | N/A | N/A | Y |
| Gaieski DF, Agarwal AK, Mikkelsen ME, Drumheller B, Cham Sante S, Shofer FS, et al. 2017. | Y | Y | Y | Y | N/A | Y | Y | N/A | N/A | N/A | Y |
| Gray A, Ward K, Lees F, Dewar C, Dickie S, McGuffie C. 2013. | Y | Y | Y | Y | N | Y | Y | N/A | N/A | N/A | Y |
| Kang MJ, Shin TG, Jo IJ, Jeon K, Suh GY, Sim MS, et al. 2012. | N | Y | Y | Y | N | Y | Y | N/A | N/A | N/A | Y |
| Le Conte P, Thibergien S, Obellianne JB, Montassier E, Potel G, Roy PM, et al. 2017. | Y | Y | Y | N | N | Y | Y | N/A | N/A | N/A | Y |
| Morr M, Lukasz A, Rubig E, Pavenstadt H, Kumpers P. 2017. | Y | Y | Y | Y | Y | Y | Y | N/A | N/A | N/A | Y |
| Shin TG, Jo IJ, Choi DJ, Kang MJ, Jeon K, Suh GY, et al. 2013. | Y | Y | Y | Y | N | Y | Y | N/A | N/A | N/A | Y |
| % | 69.23 | 100.0 | 100.0 | 76.92 | 38.46 | 100.0 | 100.0 | 0.0 | 0.0 | 0.0 | 100.0 |

#### Table: Quasi-Experimental Study

| **Citation** | **Q1** | **Q2** | **Q3** | **Q4** | **Q5** | **Q6** | **Q7** | **Q8** | **Q9** |
| --- | --- | --- | --- | --- | --- | --- | --- | --- | --- |
| Bruce HR, Maiden J, Fedullo PF, Son Chae K. 2015. | Y | N | Y | Y | Y | N/A | Y | Y | Y |
| Casserly B, Baram M, Walsh P, Sucov A, Ward NS, Levy MM. 2011. | Y | Y | Y | Y | Y | N/A | Y | Y | Y |
| Delawder JM, Hulton L. 2020. | Y | Y | Y | Y | Y | N/A | Y | Y | Y |
| Grek A, Booth S, Festic E, Maniaci M, Shirazi E, Thompson K, et al. 2017. | Y | Y | Y | Y | Y | N/A | Y | Y | Y |
| Hayden GE, Tuuri RE, Scott R, Losek JD, Blackshaw AM, Schoenling AJ, et al. 2016. | Y | Y | Y | Y | Y | N/A | Y | Y | Y |
| Jeon K, Shin TG, Sim MS, Suh GY, Lim SY, Song HG, et al. 2012. | Y | Y | Y | Y | Y | N/A | Y | Y | Y |
| Kuttab HI, Sterk E, Rech MA, Nghiem T, Bahar B, Kahn S. 2016. | Y | Y | Y | Y | Y | N/A | Y | Y | Y |
| Lorenzo MP, MacConaghy L, Miller CD, Meola G, Probst LA, Pratt B, et al. 2018. | Y | Y | Y | Y | Y | N/A | Y | Y | Y |
| Machado SM, Wilson EH, Elliott JO, Jordan K. 2018. | Y | Y | Y | Y | Y | N/A | Y | Y | Y |
| McColl T, Gatien M, Calder L, Yadav K, Tam R, Ong M, et al. 2017. | Y | Y | Y | Y | Y | N/A | Y | Y | Y |
| McDonald CM, West S, Dushenski D, Lapinsky SE, Soong C, van den Broek K, et al. 2018. | Y | Y | Y | Y | Y | N/A | Y | Y | Y |
| Nguyen HM, Schiavoni A, Scott KD, Tanios MA. 2012. | Y | Y | Y | Y | Y | N/A | Y | Y | Y |
| Papali A, Eoin West T, Verceles AC, Augustin ME, Nathalie Colas L, Jean-Francois CH, et al. 2017. | Y | Y | Y | Y | Y | N/A | Y | Y | Y |
| Ruttanaseeha W, Ienghong K, Apiratwarakul K, Bhudhisawasdi V, Hurnmek S, Gaysonsiri D. 2020. | Y | Y | Y | Y | Y | N/A | Y | Y | Y |
| Singer AJ, Taylor M, LeBlanc D, Williams J, Thode Jr HC. 2014. | Y | Y | Y | Y | Y | N/A | Y | Y | Y |
| Sivayoham N, Rhodes A, Jaiganesh T, van Zyl Smit N, Elkhodhair S, Krishnanandan S. 2012. | Y | Y | Y | Y | Y | N/A | Y | Y | Y |
| Viale P, Tedeschi S, Scudeller L, Attard L, Badia L, Bartoletti M, et al. 2017. | Y | N | Y | Y | Y | N/A | Y | Y | Y |
| Wang Z, Xiong Y, Schorr C, Dellinger RP. 2013. | Y | Y | Y | Y | Y | N/A | Y | Y | Y |
| Whitfield PL, Ratliff PD, Lockhart LL, Andrews D, Komyathy KL, Sloan MA, et al. 2020. | Y | Y | Y | Y | Y | N/A | Y | Y | Y |
| Yarbrough N, Bloxam M, Priano J, Louzon Lynch P, Hunt LN, Elfman J. 2019. | Y | N | Y | Y | Y | N/A | Y | Y | Y |
| % | 100.0 | 80.76 | 100.0 | 100.0 | 100.0 | 0.0 | 100.0 | 100.0 | 100.0 |

#### Table: Randomized Controlled Trial

| **Citation** | **Q1** | **Q2** | **Q3** | **Q4** | **Q5** | **Q6** | **Q7** | **Q8** | **Q9** | **Q10** | **Q11** | **Q12** | **Q13** |
| --- | --- | --- | --- | --- | --- | --- | --- | --- | --- | --- | --- | --- | --- |
| Andrews B, Semler MW, Muchemwa L, Kelly P, Lakhi S, Heimburger DC, et al. 2017. | Y | N | Y | N | N | Y | Y | Y | Y | Y | Y | Y | U |
| % | 100.0 | 0.0 | 100.0 | 0.0 | 0.0 | 100.0 | 100.0 | 100.0 | 100.0 | 100.0 | 100.0 | 100.0 | 0.0 |
