# Supplementary material for: Early fluid bolus in adults with sepsis in the emergency department: a systematic review, meta-analysis and narrative synthesis
Source: BMC Emerg Med. 2022 Jan 11;22:3. doi: 10.1186/s12873-021-00558-5 (PMC8753824; doi:10.1186/s12873-021-00558-5)
Supplement: Supplementary file 3 — Additional file 3. [file 12873_2021_558_MOESM3_ESM.docx]

**Additional File 3: Characteristics of Included Studies**

Table: Characteristics of Included Studies - Quasi-Experimental Study Form

| **Study** | **Country** | **Setting/context** | **Participant characteristics** | **Groups** | **Outcomes measured** | **Main description of results** | |
| --- | --- | --- | --- | --- | --- | --- | --- |
| Delawder JM, Hulton L. 2020. | USA | ED- 238 bed community hospital | Patients ≥ 18 yrs presenting to ED with clinical indications of sepsis, severe sepsis and septic shock | Intervention- Patients with sepsis presenting to ED after implementation of ED sepsis alert, educational intervention Control- Patients with sepsis presenting to ED before implementation of ED sepsis alert, educational intervention | Mortality rate; time to 3 and 6hr bundle components- blood cultures, antibiotics, collection of first and second lactate, and if indicated fluid resuscitation | The intervention improved bundle compliance and mortality in patients with sepsis who presented to ED | |
| Hayden GE, Tuuri RE, Scott R, Losek JD, Blackshaw AM, Schoenling AJ, et al. 2016. | USA | ED- urban academic hospital | Patients ≥ 18 yrs of age presenting to ED with clinical findings consistent with suspected sepsis, and/or patients meeting 2 or more criteria for systemic inflammatory response | Intervention- Patients with sepsis presenting to ED after implementation of electronic sepsis triage alert and SWAT protocol Control- Patients with sepsis presenting to ED before implementation of electronic sepsis triage alert and SWAT protocol | Specific treatment intervals- door to intravenous fluid bolus; door to antibiotics; door to admit order; length of stay in ED, ICU and hospital; overall mortality (in-hospital and hospice transfer) | Time to intravenous fluid bolus and antibiotics decreased significantly after implementation of electronic sepsis triage alert and SWAT protocol | |
| Jeon K, Shin TG, Sim MS, Suh GY, Lim SY, Song HG, et al. 2012. | South Korea | ED- 1960 bedded tertiary referral hospital | Patients ≥ 18 yrs of age presenting to ED with diagnosis of severe sepsis or septic shock | Intervention- Patients with sepsis presenting to ED after implementation of educational intervention and EGDT protocol Control- Patients with sepsis presenting to ED before implementation of educational intervention and EGDT protocol | Management of patients evaluated using interventions during the first 6hrs following diagnosis of sepsis: adequate initial fluid challenge (500-1000mL over 30 min and repeated based on response and tolerance; if non-responsive vasopressor initiation; CVP and SvO2 monitoring within 2 hrs; antibiotic administration within 4hrs; use of vasopressors, inotropes and packed red cells within 6 hrs. | 3 - month intervention of educational program and EGDT protocol improved compliance with resuscitation bundle component | |
| Bruce HR, Maiden J, Fedullo PF, Son Chae K. 2015. | USA | 2 EDs with combined bed total of 37 in a tertiary academic hospital | Adult patients presenting to ED with an International Classification of Diseases ninth revision discharge diagnosis of severe sepsis or septic shock | Before protocol- Patients admitted during the pre-protocol phase Transition- Patients admitted during the implementation of protocol After protocol- Patients admitted during the post-protocol phase | Time to initial antibiotic administration; in-hospital mortality rate; in-hospital mortality rate predictors; length of hospital stay; compliance with other surviving sepsis bindle elements- serum lactate measurement, blood culture before antibiotic administration, fluid administration ≥ 30 mL/kg within 3 hrs [if patient had hypotension or lactate level ≥ 4 mmol/L] | Implementation of ED Nurse-led sepsis protocol improved compliance to nearly 100% with lactate measurement, blood cultures before antibiotic administration, which nurses initiated independently, however, interventions requiring collaboration between 2 or more health care professional such as administration of antibiotics and fluid bolus had sub-optimal compliance. | |
| Casserly B, Baram M, Walsh P, Sucov A, Ward NS, Levy MM. 2011. | USA | ED- urban tertiary hospital | Patients presenting to ED with a real or suspected infection with either hypotension after 30mL/kg fluid bolus or a lactate ≥ 4mmol/L | Intervention- Patients with sepsis presenting to ED after the implementation of sepsis intervention program with a collaborative approach between ED and ICU Control- Patients with sepsis presenting to ED before the implementation of sepsis intervention program with a collaborative approach between ED and ICU | Time from admission to ED to catheter insertion; time to fluid administration; vasopressors and antibiotics and time to transfer from ED to MICU | Use of a collaborative approach sepsis protocol decreased time of administration of antibiotics, vasopressors and transfer to MICU, but showed no statistically significant difference in time to fluid bolus, and time to catheter insertion. However, both these elements showed an overall reduction in time in the intervention group. | |
| Grek A, Booth S, Festic E, Maniaci M, Shirazi E, Thompson K, et al. 2017. | USA | ED- 304 bedded tertiary academic medical centre | Patients discharged with the diagnosis code for severe sepsis or septic shock | Baseline- Patients who presented to ED before implementation of PDSA interventions First goal achieved- patients who presented to ED after implementation of first PDSA intervention (major intervention- creation of multidisciplinary sepsis and shock response team) Second goal achieved- patients who presented to ED after implementation of first and second PDSA intervention (major intervention- multiple communication and education tools created) | Initial and re-measurement of lactate; blood culture prior to antibiotics; antibiotics administration within first 3 hrs; administration of fluid bolus 30mL/kg; placement of central line if lactate ≥ 4 mmol/L; measurement of CVP and SCvO2; in-hospital mortality. | PDSA interventions led to significant increase in compliance with bundle elements. | |
| Kuttab HI, Sterk E, Rech MA, Nghiem T, Bahar B, Kahn S. 2016. | USA | ED- 535 bedded academic medical centre | Patients ≥ presenting to ED with severe sepsis or septic shock with documented hypoperfusion (lactate level ≥ 4mm0l/L) | Intervention- Pre-call, patients admitted to ED after addition of lactate ≥ 4 mmol/L to the critical laboratory's result call list Control- Post-call, patients admitted to ED prior to addition of lactate ≥ 4 mmol/L to the critical laboratory's result call list | Time to administration of antibiotics and fluids; total fluid volume intake; vasopressor requirement; hydrocortisone and inotrope use; number of vasopressors; 30 and 90-day mortality; ICU and hospital length of stay | Intervention did not result in a difference in time to administration of antibiotics and fluids, volume of fluid administered between both groups. | |
| Lorenzo MP, MacConaghy L, Miller CD, Meola G, Probst LA, Pratt B, et al. 2018. | USA | ED- 472 bedded academic medical centre | Patients ≥ 18 yrs of age presenting to Ed with a diagnosis of sepsis as defined by 2001 consensus International Sepsis Definitions Conference | Intervention- Patients with sepsis presenting to ED after implementation of combination antibiotic bag containing 2 g cefepime and 1 g vancomycin in 1000 mL 0.9% sodium chloride to be administered over 1 hour using central or peripheral intravenous access and education. Control- Patients with sepsis presenting to ED before implementation of combination antibiotic bag containing 2 g cefepime and 1 g vancomycin in 1000 mL 0.9% sodium chloride to be administered over 1 hour using central or peripheral intravenous access and education. | Proportion receiving at least 2 antibiotics and 30mL/kg fluid within first 3 hrs; volume of fluids administered; time of administration of at least 2 antibiotics. | Intervention was associated with significant increase in the percentage of patients who received at least 2 antibiotics and 30mL/kg fluid within the first 3 hrs. | |
| Machado SM, Wilson EH, Elliott JO, Jordan K. 2018. | USA | ED- tertiary hospital | Patients ≥ 18 yrs of age presenting to ED with suspected sepsis and have a confirmed discharge diagnosis of sepsis | Intervention- Patients with sepsis presenting to ED after implementation of activation of sepsis alert and electronic ICU cart following ED physician sepsis bundle orders (an electronic intensive medical unit cart directly links the patient to an intensivist [located elsewhere in the hospital] who will review the care and place additional orders as needed) Control- Patients with sepsis presenting to ED before implementation of activation of sepsis alert and electronic ICU cart | Antibiotic administration and blood culture within first 3 hrs; lactate measurement within 6 hrs; normal saline within 6 hrs; ED and hospital length of stay; discharge disposition; readmission within 30 days; mortality and hospital costs | Intervention improved adherence to Surviving sepsis bundles but not mortality or healthcare cost. | |
| McColl T, Gatien M, Calder L, Yadav K, Tam R, Ong M, et al. 2017. | Canada | 2 EDs from the tertiary academic hospital | Patients ≥ 18 yrs of age presenting to ED who meet the criteria for suspected severe or serious infection and two or more of the SIRS criteria based on the STEP protocol | Intervention- Patients with sepsis presenting to ED after implementation of "Targeted Sepsis" intervention (with multiple interventions: new triaging tools with flagging system; protocolised ED destination from triage to monitored bed; nurse initiated fluid resuscitation; multiple educational tools and training Control- Patients with sepsis presenting to ED before implementation of "Targeted Sepsis" intervention | 30-day all-cause mortality; time from triage to physician assessment; time to intravenous fluids; time to antibiotic administration; lactate clearance; use of vasopressor/inotropic medication and ICU admission. | Intervention resulted in significant use of sepsis protocol, decrease in mortality, reduction in time to physician assessment; first fluid bolus and antibiotic administration. | |
| McDonald CM, West S, Dushenski D, Lapinsky SE, Soong C, van den Broek K, et al. 2018. | Canada | 35 bedded ED- teaching hospital | Patients presenting to ED who received an ICD-10-CA code for sepsis, septicemia and all infections. | Intervention- Patients with sepsis presenting to ED after implementation of SNAP algorithm (algorithm including patient inclusion criteria, interventions and time targets for clinicians; electronic order sets specific to sepsis care; nursing medical directives; education) Control- Patients with sepsis presenting to ED before implementation of SNAP algorithm | Time to assessment by ED physician, lactate measurement, blood culture collection, fluid and antibiotic administration; ICU admission; ICU length of stay; mortality | Interventions improved time to antibiotics and fluids | |
| Nguyen HM, Schiavoni A, Scott KD, Tanios MA. 2012. | USA | 47 bedded ED- community based teaching hospital | Patients ≥ 18 yrs of age presenting to ED with an admission diagnoses (ICD-9: 995.91 for Sepsis, 995.92 for Severe Sepsis and 785.52 for Septic Shock) of sepsis or septic shock admitted from the ED to the ICU. | Intervention- Patients with sepsis presenting to ED after implementation of Sepsis Education Program Control- Patients with sepsis presenting to ED before implementation of Sepsis Education Program | Total quantity of IV fluids administered within the first 3 hrs; appropriate antibiotic administration in ED; hospital mortality; hospital length of stay. | Intervention resulted in early aggressive and appropriate fluid resuscitation and administration of antibiotics | |
| Ruttanaseeha W, Ienghong K, Apiratwarakul K, Bhudhisawasdi V, Hurnmek S, Gaysonsiri D. 2020. | Thailand | ED- University teaching hospital | Patients ≥ 18 yrs of age presenting to ED who fulfilled the criteria for sepsis diagnosis. | Intervention- Patients with sepsis presenting to ED after the implementation of SWAT (Sepsis Workup And Treatment) protocol Control- Patients with sepsis presenting to ED before the implementation of SWAT (Sepsis Workup And Treatment) protocol | Administration of antibiotics within one hour; measurement of lactate, obtaining blood culture, IV fluid administration within 3 hrs; mortality | Intervention improved compliance with measurement of lactate, obtaining blood cultures and adequate fluids for patients with low blood pressure. | |
| Singer AJ, Taylor M, LeBlanc D, Williams J, Thode Jr HC. 2014. | USA | ED- academic tertiary medical centre | Patients ≥ 18 yrs of age presenting to ED with a suspected infection, met minimum of 2 physiological criteria for SIRS, [consented to participate in the study (for the intervention arm)]. | Intervention- Patients with sepsis presenting to ED after the implementation of ED bedside Point of Care Lactate Control- Patients with sepsis presenting to ED before the implementation of ED bedside Point of Care Lactate | Ordering and reporting of central lactate levels; IV catheter insertion; IV fluid administration; antibiotic administration; time of decision to admit; actual time of admission; in-hospital mortality. | Intervention resulted in significant reduction in time to test results, IV fluid administration; ICU admission rates and in-hospital mortality. | |
| Viale P, Tedeschi S, Scudeller L, Attard L, Badia L, Bartoletti M, et al. 2017. | Italy | ED-1420 bedded teaching hospital | Patients ≥ 18 yrs of age presenting to ED with severe sepsis or septic shock based on Surviving Sepsis guidelines | Intervention- Patients with sepsis presenting to ED after the implementation of Sepsis Team with 13 Infectious Diseases specialists who evaluated patients at bedside within 1 hour. Control- Patients with sepsis presenting to ED before the implementation of Sepsis Team with 13 Infectious Diseases specialists | All-cause 14- day mortality; compliance with all items in Surviving Sepsis bundle; attempt to etiological diagnosis; proportion of patients with documented causative agents; appropriateness of initial antibiotic therapy. | Intervention significantly improved lactate measurement; fluid resuscitation; drawing blood cultures; administration of first antibiotic within 3 hours. | |
| Wang Z, Xiong Y, Schorr C, Dellinger RP. 2013. | China | ED- University affiliated hospital | Patients presenting to ED meeting the criteria for severe sepsis or septic shock | Intervention- Patients with sepsis presenting to ED after the implementation of sepsis bundles Control- Patients with sepsis presenting to ED before the implementation of sepsis bundles | Compliance with sepsis bundles- lactate measurement, CVP measurement, ScvO2 measurement within 6hrs; blood culture prior to antibiotics; antibiotics administered within 3 hrs; 20mL/kg fluids administered followed by vasopressors; glucose control; median IPP <30cm H2O; mortality; reasons given for not achieving target goals. | Intervention improved compliance with sepsis bindle elements and mortality. Barriers to implementation identified were knowledge, attitude and behavioural. | |
| Whitfield PL, Ratliff PD, Lockhart LL, Andrews D, Komyathy KL, Sloan MA, et al. 2020. | USA | 35 bedded ED- tertiary hospital | Patients ≥ 18yrs of age presenting to Ed who meet the criteria for Centres for Medicare and Medicaid definition of severe sepsis or septic shock. | Intervention- Patients with sepsis presenting to ED after the implementation of the Adult Code Sepsis Protocol Control- Patients with sepsis presenting to ED before the implementation of the Adult Code Sepsis Protocol | Overall compliance with SEP-1 Perfect Score Attainment (PSA)- completion of all 3 hr (blood culture before antibiotics; lactate measurement; antibiotic administration) and 6 hr (vasopressor administration; repeat lactate; repeat assessment) bundle element completion; all-cause-in-hospital mortality; economic outcomes- total cost of hospitalisation; direct variable cost per case. | Implementation resulted in significant improvement in SEP-1 PSA | |
|  | | | | | | |  |
|  | | | | | | |  |
| Yarbrough N, Bloxam M, Priano J, Louzon Lynch P, Hunt LN, Elfman J. 2019. | USA | ED- quaternary community hospital | Patients ≥ 18 yrs of age who presented to ED, met criteria for generating an initial sepsis alert, severe sepsis or septic shock. | Intervention- Patients with sepsis presenting to ED after the implementation of the intervention (incorporation of pharmacists as standard part of the multidisciplinary sepsis response team) Control- Patients with sepsis presenting to ED after the implementation of the intervention | Completion rates of SEP-1 3hr and 6hr bundles; time to completion of fluid administration; blood cultures; antibiotic administration | Intervention showed no significant difference in 3hr and 6hr bundle compliance but showed decrease in time to the individual elements of the bundle. | |
| Papali A, Eoin West T, Verceles AC, Augustin ME, Nathalie Colas L, Jean-Francois CH, et al. 2017. | Haiti | ED- resource-limited 80- bedded community referral hospital | Patients ≥ 17yrs of age presenting to ED with severe sepsis or septic shock. | Intervention- Patients with sepsis presenting to ED after the implementation of adapted version of WHO severe sepsis protocol Control- Patients with sepsis presenting to ED before the implementation of adapted version of WHO severe sepsis protocol | Physician recognition of sepsis; volume of IVF administered; time from triage to fluid administration; time from triage to antibiotic administration; time from triage to second vital signs; mortality. | Intervention showed no significant difference in mortality; time to fluid administration and antibiotic administration; proportion of antibiotic administration; intervention improved volume of fluids administered; repeat vital signs; proportion of point of care lactate and Chest Xray. | |
| Bond CM, Djogovic D, Villa-Roel C, Bullard MJ, Meurer DP, Rowe BH. 2013. | Canada | ED- University hospital | Adults ≥ 16 yrs of age, presenting to ED, receiving ICD Code 10 for sepsis, septicaemia, severe sepsis. | Intervention- patients with Severe sepsis/Septicaemia for whom the electronic Clinical Practice Guideline (eCPG) was used based on qualifying criteria of hypotension, elevated heart rate, or increased serum lactate.  Control- patients diagnosed with ICD code 10 for sepsis, septicaemia and severe sepsis in whom eCPG was not used. | Exposure- electronic Clinical Practice Guideline Variable measured- serum lactate, blood culture before antibiotic administration, antibiotic administration within 3 hrs, delivery of 20mL/kg intravenous fluids, vasopressors for non-fluid responsive patients, measurement of CVP and CvSO2. | ‘‘Protocolized’’ care in the form of an eCPG improved intermediate outcomes by decreasing the time required for laboratory tests and increasing the completion of recognized important interventions. | |

# Table: Characteristics of Included Studies - Randomized Controlled Trial Form

| **Study** | **Country** | **Setting/context** | **Participant characteristic** | **Groups** | **Outcomes measured** | **Description of main results** |
| --- | --- | --- | --- | --- | --- | --- |
| Andrews B, Semler MW, Muchemwa L, Kelly P, Lakhi S, Heimburger DC, et al. 2017. | Zambia | ED - 1500 bedded hospital | Patients presenting to ED aged 18 years or older if they had (1) sepsis (defined as suspected infection plus ≥2 systemic inflammatory response syndrome criteria) and hypotension (defined as systolic blood pressure ≤90mmHg or mean arterial pressure ≤65mmHg). | Intervention - Sepsis protocol group- initial 2 L intravenous fluid bolus was administered within 1 hr, additional 2L over subsequent 4 hrs. Control- Usual care group- treating clinicians determined intravenous fluid bolus | In- hospital mortality; volume of fluids administered within 6, 24 and 72 hours. | In the intervention group, patients received 4L or greater of intravenous fluid between ED registration and 6 hours. In adults with sepsis and hypotension, most of whom had been diagnosed with HIV, in a resource-limited setting, a protocol for early resuscitation with intravenous fluid boluses and vasopressors increased mortality compared with usual care. |

# Table: Characteristics of Included Studies - Cohort Study Form

| **Study** | **Country** | **Setting/context** | **Participant characteristic** | **Groups** | **Outcomes measured** | **Description of main results** |
| --- | --- | --- | --- | --- | --- | --- |
| Baldwin LN, Smith SA, Fender V, Gisby S, Fraser J. 2008. | United Kingdom | ED in a district general hospital | All patients that presented with severe sepsis with a low blood pressure non-responsive to fluid alone and/or a lactate of over 4 mmol/L were included | Compliance with each element within the resuscitation bundle | i) Appropriate fluid challenge (20 ml/kg crystalloid or 7 ml/kg colloid)  ii) Blood culture before antibiotic administration  iii) First dose of an appropriate antibiotic given within 3 h  iv) Measurement of arterial lactate  v) Site a central venous catheter if remains hypotensive after fluid challenge  vi) Measurement and use of central venous pressure to target further fluid therapy  vii) Use of a pressor (Noradrenaline) to achieve target MAP  viii)Measurement and use of central venous oxygen saturation to guide inotrope use | The overall compliance rate was low and varied from complete (arterial lactate) to around 50% for fluid challenge, antibiotic administration and appropriate use of central venous catheter. |
| Gaieski DF, Agarwal AK, Mikkelsen ME, Drumheller B, Cham Sante S, Shofer FS, et al. 2017. | USA | ED in academic, level 1 trauma centre | Patients with severe sepsis presenting to ED | Three severe sepsis cohorts were used to assess outcomes: 1) those who did not qualify for protocolized care; 2) those who qualified for protocolized care and received it; 3) those who qualified for protocolized care and did not receive it. ED crowding was measured using the ED occupancy rate. | Primary outcomes measured were: 1) time to administration of IVF; 2) time to administration of antibiotics; 3) whether protocolized care was initiated, and 4) in-hospital mortality. | Increased ED crowding significantly delays time to initial administration of intravenous fluids and antibiotics and decreases the implementation of protocolized care but, contrary to our hypothesis, does not significantly impact mortality. |
| Faine BA, Noack JM, Wong T, Messerly JT, Ahmed A, Fuller BM, et al. 2015. | USA | ED- 711 bedded academic medical centre, transferring hospitals- rural critical care access and local community hospitals | Patients ≥ 18 years presenting to ED diagnosed with severe sepsis or septic shock based on International Classification of Diseases, 9th Revision, Clinical Modification criteria. | Group1- transfer patients who arrived at the academic medical centre ED after receiving care in a local community hospital  Group 2- patients who directly presented to the academic medical centre ED | Assess whether inter-hospital transfer delays administration of antibiotics and other surviving sepsis bundle elements; length of stay; 28-day mortality | Inter-hospital transfer leads to significant delays in antibiotic and fluid bolus administration. |
| De Groot B, Struyk B, Najafi R, Halma N, Pelser L, Vorst D, et al. 2017. | Netherlands | ED- 2 University medical centres | Patients ≥ 16 yrs of age with a suspected infection and a Manchester triage category for those with urgent medical needs, who received antibiotics and subsequently admitted to the hospital. | Patients with early or severe sepsis presenting to ED after implementation of the sepsis quality improvement program (introduction of screening procedure, education and training) | Time to antibiotics within first 3 hrs; appropriateness of antibiotics (for culture positive vs negative patients); blood cultures obtained prior to antibiotic administration; accuracy of suspected source of infection; lactate measurement; MAP within first 6 hrs; ICU consultation for severe sepsis/septic shock; minimum 1.5 L IV fluids administered in case of shock; any amount of fluid administered if there is no shock; unanticipated transfer to ICU. | Full compliance with all elements of surviving sepsis bundle was associated with lower in-hospital mortality. |
| Deis AS, Whiles BB, Brown AR, Satterwhite CL, Simpson SQ. 2018. | USA | ED- University hospital | Patients ≥ 18 yrs of age presenting to ED who received an ICD-10 diagnosis code for acute infection and given antibiotic within first 8 hrs. After meeting this inclusion criteria, patients were retained if they met case definitions for severe sepsis: 1) a 995.92 ICD-9 diagnosis code for severe sepsis or 2) documented presence of severe infection plus two or more sites of organ dysfunction. | Group 1- patients with 995.92 code  Group 2- patients without 995.92 code | Administration of minimum 30mL/kg fluids within 3 hrs if patient was hypotensive or lactate level ≥ 4 mmol/L; administration of antibiotics within first 3 hrs; serum lactate measurement; blood culture | All patients regardless of receiving or not receiving 995.92 Codes were unlikely to receive treatment within first 3 hrs; however, those with the code received treatment more often than those without the code. |
| Gray A, Ward K, Lees F, Dewar C, Dickie S, McGuffie C. 2013. | Scotland | ED- 20 mainland Scottish hospitals | Patients ≥ 16 yrs of age presenting to ED who met the sepsis criteria: 1) suspected or confirmed infection within 2 days of presentation; 2) presence of 2 of the following physiological criteria: temperature > 38.3 deg Celsius or < 36 deg Celsius, respiratory rate >20/min, white cell count >12 000/ml or <4000/ml or >10% immature forms; acutely altered mental status; systolic blood pressure <90 mm Hg; and blood glucose >7.7 mmol/l (in the absence of diabetes) | Group 1- Patients who met were hypotensive Group 2- Patients who had serum lactate ≥ 4mmol/L  Group 3- Patients who were hypotensive and had serum lactate ≥ 4mmol/L | Measurement of lactate, blood cultures, CVP and SvO2 within first 6 hrs; antibiotic administration within first 3 hrs; administration of fluid bolus up to 20mL/kg within first 6 hrs; in-hospital mortality; ICU admission; length of stay | Fluid resuscitation component of the surviving sepsis bundle was found to be low. Antibiotic administration, lactate measurement and blood cultures also had low compliance. |
| Le Conte P, Thibergien S, Obellianne JB, Montassier E, Potel G, Roy PM, et al. 2017. | France | ED- 2 teaching hospitals | Patients ≥ presenting to ED with severe sepsis as defined by 2001 consensus conference. | Not applicable- no change to clinical management and had no written guidelines | Admission to ICU; hospital length of stay; mortality; obtaining blood culture; antibiotic administration within first 3 hrs; fluid administration ≥ 30mL/kg within first 3 hrs | There is poor compliance with the surviving sepsis bundles and delay in time of diagnosis of sepsis. |
| Morr M, Lukasz A, Rubig E, Pavenstadt H, Kumpers P. 2017. | Germany | ED- Multi disciplinary University hospital, managed by Department of Internal Medicine | All medical patients ≥ 18 yrs presenting to ED screened for signs of infection | Non-SIRS- Patients classified as non-SIRS (infection)Sepsis- Patients classified as sepsis  Severe sepsis- Patients classifies as severe sepsis | In-hospital mortality; detection of septic focus; antibiotic administration; adequate fluids | A significant proportion of patients who met the criteria for definition of sepsis were not recognised. Non-recognition of sepsis was not associated with adequacy of initial therapy in ED. |
| Shin TG, Jo IJ, Choi DJ, Kang MJ, Jeon K, Suh GY, et al. 2013. | South Korea | ED- 1960 bedded tertiary referral hospital | Patients ≥ 18 yrs of age presenting to ED with severe sepsis or septic shock with a lactate ≥ 4 mmol/L | Group 1- Patients who presented to ED during low-crowding (ED occupancy state calculated from the number of patients in the ED at the time of triage.  Group 2- Patients who presented to ED during intermediate -crowding  Group 3- Patients who presented to ED during high-crowding | Overall compliance of the entire resuscitation bundle; completion rate of each intervention- lactate measure, blood culture, antibiotic administration, fluid challenge, CVP ≥ 8 mmHg, MAP ≥ 65 mmHg, ScvO2 ≥ 70%; number of bundle elements completed; ED, ICU and in-hospital length of stay; in-hospital mortality. | ED crowding was associated with lower compliance with the entire resuscitation bundle. |
| Kang MJ, Shin TG, Jo IJ, Jeon K, Suh GY, Sim MS, et al. 2012. | South Korea | ED- 1960 bedded tertiary referral hospital | Patients ≥ 18 yrs and older presenting to ED with severe sepsis or septic shock and lactate ≥ 4 mmol/L | Group 1- High compliance group- Patients with completion of 6 or 7 out of 7 interventions of the sepsis protocol bundle within first 6 hrs.  Group 2- Low compliance group- Patients with completion of 0-5 out of 7 interventions of the sepsis protocol bundle within first 6 hrs. | Potential factors associated with higher compliance with resuscitation bundle; proportion of compliance with resuscitation bundle elements within 6 hrs- serum lactate measurement; antibiotic administration; IV fluid challenge; MAP ≥ 65 mmHg achieved; CVP 8-12 mmHg achieved; CvO2 ≥ 70% achieved; ED, ICU, hospital length of stay; in-hospital mortality | Rate of compliance with 6 hr resuscitation bundle was significantly lower in patients with cryptic shock, higher serum lactate level, or ones without hyperthermia. High rates of compliance was associated with nurses with > 3yrs of clinical experience, senior residents and board-certified emergency physicians. |
