# Supplementary material for: Early fluid bolus in adults with sepsis in the emergency department: a systematic review, meta-analysis and narrative synthesis
Source: BMC Emerg Med. 2022 Jan 11;22:3. doi: 10.1186/s12873-021-00558-5 (PMC8753824; doi:10.1186/s12873-021-00558-5)

**Additional File- Funnel Plots**

1) Funnel plot of studies reporting compliance


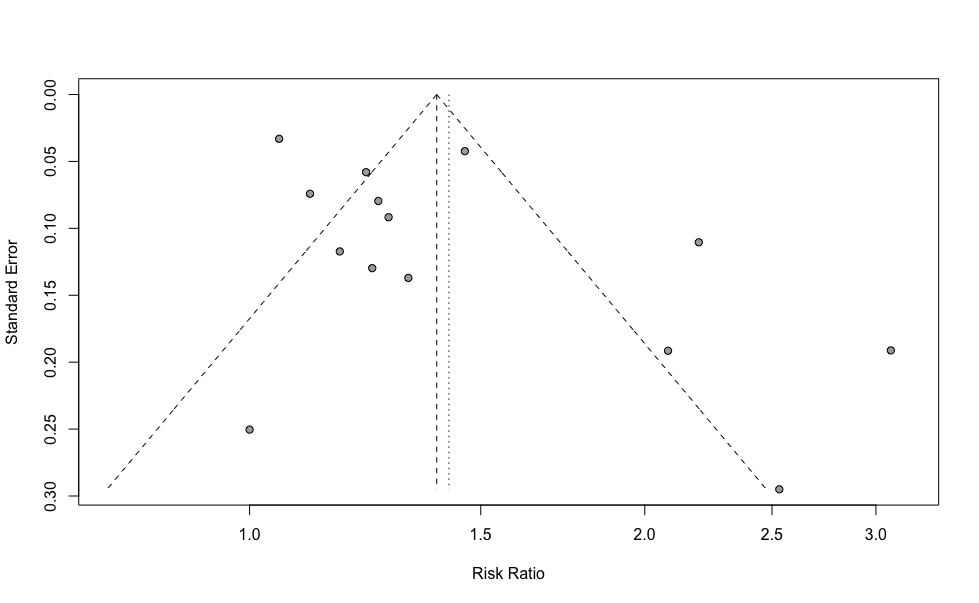


2) Funnel plot of studies reporting time to fluids


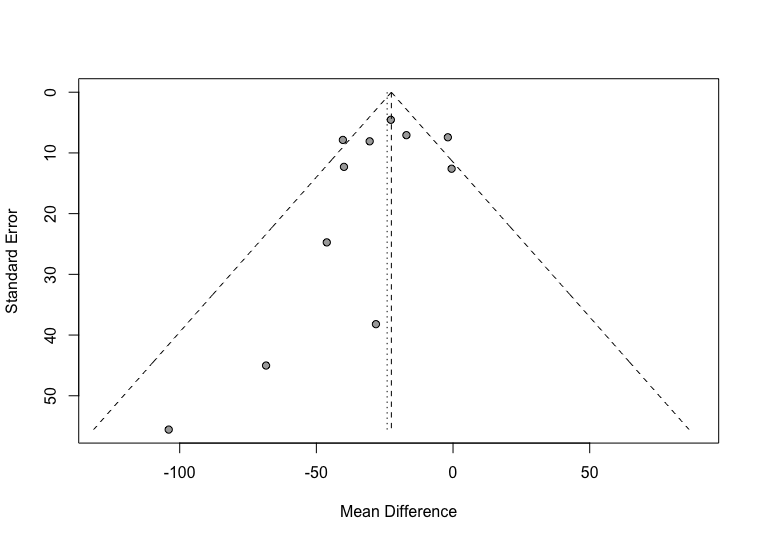


3) Funnel plot of studies reporting volume of fluids


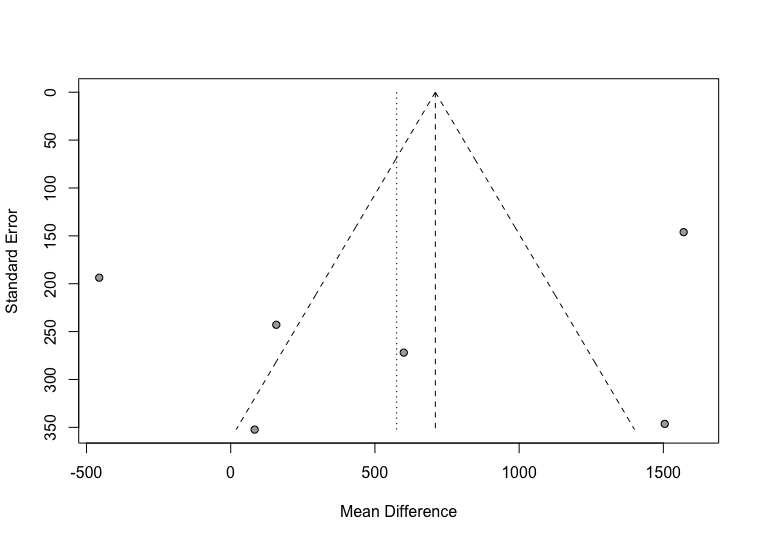

Supplement: Supplementary file 4 — Additional file 4. [file 12873_2021_558_MOESM4_ESM.docx]
