# Supplementary material for: Early fluid bolus in adults with sepsis in the emergency department: a systematic review, meta-analysis and narrative synthesis
Source: BMC Emerg Med. 2022 Jan 11;22:3. doi: 10.1186/s12873-021-00558-5 (PMC8753824; doi:10.1186/s12873-021-00558-5)
Supplement: Supplementary file 5 — Additional file 5. [file 12873_2021_558_MOESM5_ESM.docx]

**Additional File 5- Meta-regression**

| **Studies reporting Compliance with early fluid administration** | | | | | |
| --- | --- | --- | --- | --- | --- |
| Predictor | Estimated amount of residual heterogeneity  (tau^2) | Unaccounted Variability  (I^2) | Amount of heterogeneity accounted for  (R^2) | Test for residual heterogeneity  QE (df = 8) | P value |
| Mortality | 0.118 (SE = 0.066) | 90% | 0.00% | 94.22 | < .001 |
| Number of interventions | 0.083 (SE = 0.041) | 90% | 0.00% | 73.10 | < .001 |
| Year of Study Commencing | 0.087 (SE = 0.041) | 93% | 0.00% | 102.41 | < .001 |
| **Studies reporting time to intravenous fluids** | | | | | |
| Predictor | Estimated amount of residual heterogeneity  (tau^2) | Unaccounted Variability  (I^2) | Amount of heterogeneity accounted for  (R^2) | Test for residual heterogeneity  QE (df = 8) | P value |
| Mortality | 73.185 (SE = 125.541) | 58% | 45.41% | 7.25 | 0.404 |
| Number of interventions | 201.965 (SE = 170.978) | 58% | 0.00% | 23.85 | 0.005 |
| Year of Study Commencing | 186.312 (SE = 162.079) | 61% | 0.00% | 22.21 | 0.008 |
| **Studies reporting volume of intravenous fluids** | | | | | |
| Predictor | Estimated amount of residual heterogeneity  (tau^2) | Unaccounted Variability  (I^2) | Amount of heterogeneity accounted for  (R^2) | Test for residual heterogeneity  QE (df = 8) | P value |
| Mortality | 733313.953 (SE = 646040.523) | 94% | 0.00% | 74.08 | < .001 |
| Number of interventions | 181489.591 (SE = 206449.816) | 94% | 75.43% | 11.84 | 0.008 |
| Year of Study Commencing | 777743.708 (SE = 600071.761) | 94% | 0.00% | 80.28 | < .001 |
