# Supplementary material for: Early fluid bolus in adults with sepsis in the emergency department: a systematic review, meta-analysis and narrative synthesis
Source: BMC Emerg Med. 2022 Jan 11;22:3. doi: 10.1186/s12873-021-00558-5 (PMC8753824; doi:10.1186/s12873-021-00558-5)
Supplement: Supplementary file 6 — Additional file 6. [file 12873_2021_558_MOESM6_ESM.docx]

**Additional File 6- Association with year of commencing study reflecting guideline changes**

| 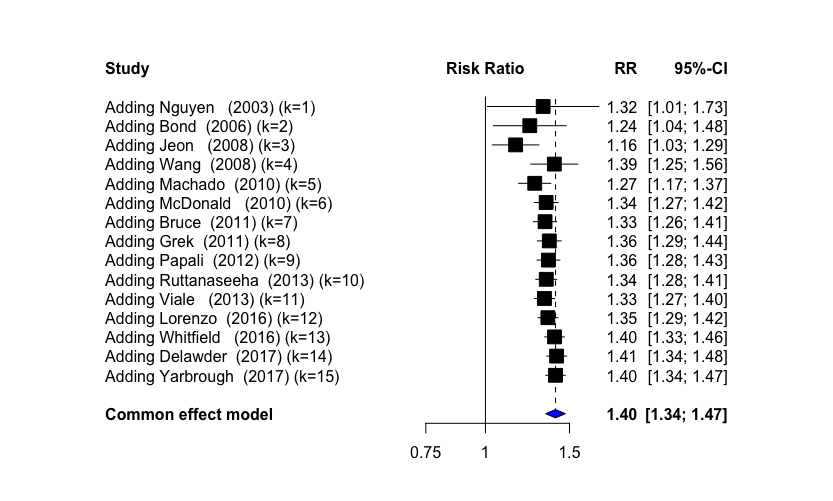 |
| --- |
| **Figure 1: Association between year study commenced and compliance with early initiation of intravenous fluid bolus** |

| 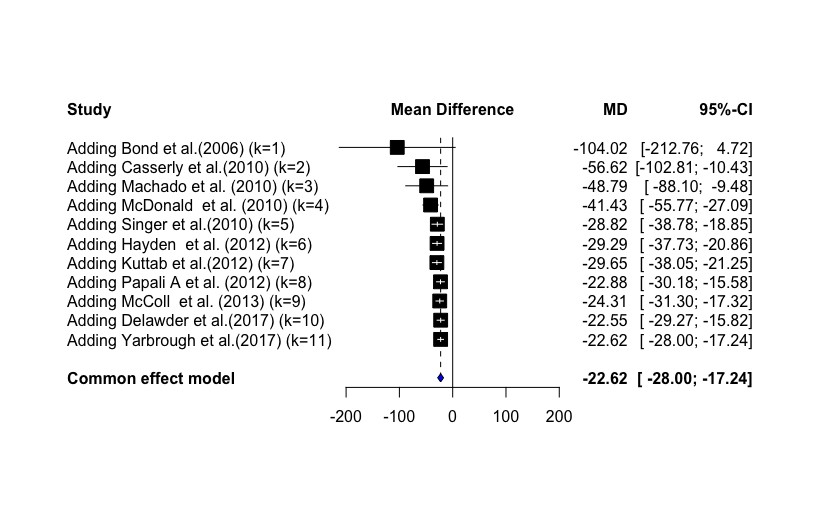 |
| --- |
| **Figure 2: Association between year of study commencing and time of initiation of first intravenous fluid bolus administration** |

| 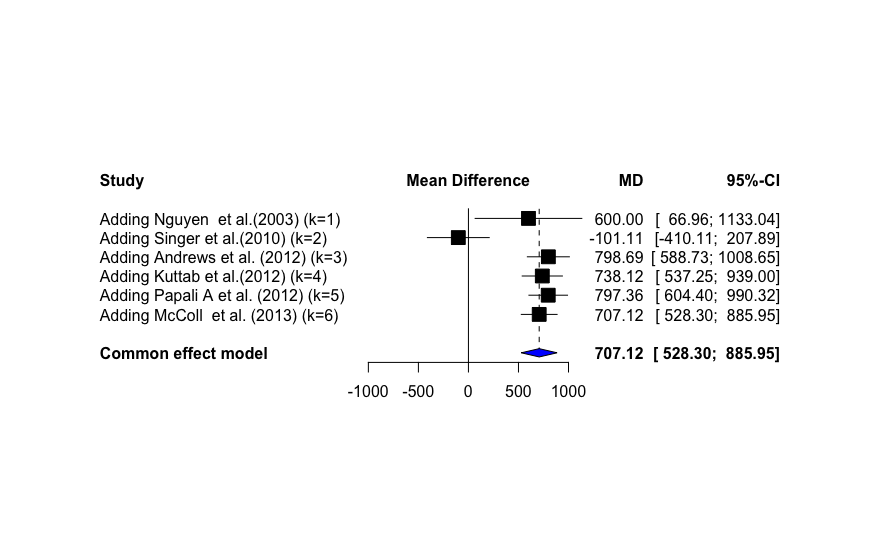 |
| --- |
| **Figure 3 Association between year of study commencing and volume of fluids administered within the protocol recommended time** |
